# Intraoperative electron radiotherapy in early invasive ductal breast cancer: 6-year median follow-up results of a prospective monocentric registry

**DOI:** 10.1186/s13058-022-01582-4

**Published:** 2022-11-23

**Authors:** Catherine Philippson, Samuel Larsen, Stéphane Simon, Christophe Vandekerkhove, Alex De Caluwe, Dirk Van Gestel, Marie Chintinne, Isabelle Veys, Filip De Neubourg, Danièle Noterman, Mirela Roman, Jean-Marie Nogaret, Antoine Desmet

**Affiliations:** 1 Department of Radiation Oncology, Jules Bordet Institute, 1000 Brussels, Belgium; 2 Department of Surgery, Jules Bordet Institute, 1000 Brussels, Belgium; 3 Department of Pathology, Jules Bordet Institute, 1000 Brussels, Belgium; 4 Department of Radiophysics, Jules Bordet Institute, 1000 Brussels, Belgium; 5 Faculté de Médecine, Université Libre de Bruxelles, 1070 Brussels, Belgium; 6 Institut Jules Bordet, 90 Rue Meylemeersch, 1070 Anderlecht, Belgium

**Keywords:** Early stage breast cancer, Accelerated partial breast irradiation (APBI), Intraoperative electron radiotherapy (IOERT)

## Abstract

**Background:**

Intraoperative electron radiotherapy (IOERT) can be used to treat early breast cancer during the conservative surgery thus enabling shorter overall treatment times and reduced irradiation of organs at risk. We report on our first 996 patients enrolled prospectively in a registry trial.

**Methods:**

At Jules Bordet Institute, from February 2010 onwards, patients underwent partial IOERT of the breast. Women with unifocal invasive ductal carcinoma, aged 40 years or older, with a clinical tumour size ≤ 20 mm and tumour-free sentinel lymph node (on frozen section and immunohistochemical analysis). A 21 Gy dose was prescribed on the 90% isodose line in the tumour bed with the energy of 6 to 12 MeV (Mobetron®-IntraOp Medical).

**Results:**

Thirty-seven ipsilateral tumour relapses occurred. Sixteen of those were in the same breast quadrant. Sixty patients died, and among those, 12 deaths were due to breast cancer. With 71.9 months of median follow-up, the 5-year Kaplan–Meier estimate of local recurrence was 2.7%.

**Conclusions:**

The rate of breast cancer local recurrence after IOERT is low and comparable to published results for IORT and APBI. IOERT is highly operator-dependent, and appropriate applicator sizing according to tumour size is critical. When used in a selected patient population, IOERT achieves a good balance between tumour control and late radiotherapy-mediated toxicity morbidity and mortality thanks to insignificant irradiation of organs at risk.

**Supplementary Information:**

The online version contains supplementary material available at 10.1186/s13058-022-01582-4.

## Introduction

The current standard of care for early stage breast cancer is breast conserving surgery (BCS) followed by adjuvant whole breast irradiation (WBI) [1]. This strategy extends the treatment time to about 1–3 weeks with classic treatment modalities [2]. This treatment time can be a cause of non-compliance to radiotherapy treatment (RT). Badakhshi et al. found that in a German cohort at a reference centre for breast cancer treatment, about 5% of patients were not compliant to adjuvant external radiotherapy treatment [3]. Half of those patients claimed that accessing the treatment centre every day of the week was logistically too difficult. Non-compliance increases dramatically in emerging and developing countries where radiotherapy centres are further apart and harder to access [4]. Yet to do without radiotherapy after BCS is not acceptable, as a number of randomized control trials (RCTs) has shown [5–8]. Multiple options are becoming available to reduce treatment duration. For example, hypofractionated WBI radiotherapy that reduces the total treatment time appears to be quickly becoming the new standard in early breast cancer [9, 10]. Accelerated partial breast irradiation (APBI) is also emerging as another option [11–16].

In the case of this study, we explore partial breast intraoperative radiotherapy (IORT), drastically reducing treatment time to a one-shot, radiotherapy treatment in the operating room.

The other major advantage of IORT is that the procedure reduces the size of irradiated breast tissue, thus significantly lowering the radiotherapy dose delivered to organs at risks (OAR). WBI, which is used as the current standard of care, is directly responsible for an increase in morbidity and non-breast cancer-related deaths mediated by an increase in the incidence of lung and heart disease proportionate to the radiotherapy dose delivered to those organs [17–20]. The intraoperative electron radiotherapy (IOERT) used in our hospital allows for the delivery of RT while entirely sparing the lungs and heart by placing a shield on the pectoral wall during the procedure.

This registry trial brings us high quality real-world data and evidence to support IOERT use in selected patients for early breast cancer. It also brings possible explanations for the disappointing results of the ELIOT IOERT RCT [21, 22].

## Methods

### Patient selection and preoperative workup

Within an ongoing prospective registry trial, patients who received IOERT as APBI were evaluated according to breast cancer survival and recurrence as primary endpoints at Institut Jules Bordet hospital in Brussels, Belgium. This study was approved by an institutional review board and included eligible patients from February 2010 until October 2019.

Female patients aged 40 years or over with biopsy proven unifocal invasive ductal carcinoma of clinical size ≤ 20 mm, and no lymph node involvement upon sentinel lymph node frozen section pathological examination were considered eligible for study inclusion. Furthermore, no restrictions were made towards molecular breast cancer subtypes as defined by histological grading, hormonal or HER2 receptor status.

On the contrary, if sentinel lymph node examination was deemed unfeasible on preoperative lymphoscintigraphy, as is the case with sole internal mammary lymph drainage, patients were excluded from our study. Patients with lymphovascular involvement and invasive lobular carcinoma on preoperative biopsy specimen were excluded. Likewise patients with a suspicion of extensive intraductal disease on preoperative MRI or core biopsy were excluded.

Every patient that presented at our institution fulfilling the above-mentioned criteria was offered IORT APBI. Every patient that undertook this treatment while fulfilling the criteria was included in the registry.

Eligible patients underwent a preoperative workup consisting of conventional mammography and echography as well as breast MRI to rule out multifocal disease. Additionally, an echography-guided and/or MRI-guided biopsy was performed as deemed necessary. All patients underwent a metastatic work-up including a chest X-ray, liver ultrasound, bone radionuclide scan and blood tests.

### Surgery and IOERT

All patients underwent a lumpectomy with sentinel lymph node excision (SLN) following our previously published protocol (Additional file 1: Appendix A). Once a first frozen section analysis of the surgically removed tumour and lymph node had confirmed a tumour-free margin ≥ 1 mm and no lymph node involvement (pN0), patients were cleared for intraoperative irradiation. In the case of a tumour bordering the pectoral aponeurosis, the aponeurosis was removed to ensure safe margins.

In some patients, final pathology revealed a positive sentinel lymph node despite consistently negative SLN results on frozen sections. If patients presented with a macro metastasis in the SLN final pathology, complete axillary node dissection was performed. In cases of pN1mic, this was deemed unwarranted. Patients presenting with micro- or macroscopic spread to the sentinel lymph node were not given additional external radiation therapy. Additionally, if there was invasive tumour on the final pathology margin, patients were offered a re-excision whenever deemed feasible.

Similarly, despite our best efforts to exclude patients with multifocal breast tumours from this study, final pathology analysis sometimes revealed patients with multifocal lesions, i.e. a second tumour adjacent to the first in the same lumpectomy piece. These patients were included in this study and reported as multifocal breast cancers.

All patients were treated with electrons generated by an IntraOp (Mobetron) dedicated mobile accelerator as detailed in our previous protocol (Additional file 1: Appendix A). The tumour was removed in one piece with a 1–2 cm safety margin. A shield was inserted on the pectoral aponeurosis. The breast tissue surrounding the tumour dissection was then sutured over the shield. A cylindrical applicator is then placed over the breast tissue. As a rule, the field diameter used was at least 40 mm bigger than the pathological tumour diameter (thus a 10 mm tumour would be treated with a 50 mm applicator diameter). The dose delivered was 21 Gy, prescribed over the 90% isodose line.

### Systemic treatments

Systemic treatments were determined according to institutional guidelines taking into consideration the tumour’s molecular subtype and the patients’ comorbidities.

### Follow-up and results assessment

All patients were seen at the clinic 10 days after surgery to assess for acute treatment toxicity. Subsequent follow-up consisted of regular check-ups and imaging according to our institutional guidelines. At the very least, all patients were scheduled for an annual mammogram, bilateral breast echography, blood sample and clinician consult. When possible, cosmetic results were assessed by the physician during the consult according to the RTOG, CTCAE v3.0 and LENT SOMA scales [23, 24].

### Overall survival and local recurrence

Follow-up time ran from the operation date to the patient’s death or last known follow-up. We systematically attempted to contact and reschedule patients that had missed appointments to minimize patients lost to follow-up.

Patients were considered to have local recurrence or distant disease as soon as pathological confirmation was obtained. Biopsy to confirm local recurrence or metastasis was always sought when necessary to guide systemic therapy.

Time to recurrence or metastasis represents the time from the IOERT to the reported event. All end-point variables reported are defined according to the DATECAN guidelines [25]. Molecular subtypes were defined according to the St-Gallen 2013 guidelines [26].

### Statistical analysis

The reference date for analysis was 01/08/2021, insofar as all patient data up to that date were included for analysis. Five-year event rates were estimated using the Kaplan–Meier estimator. Multivariate competing risk regression was used to assess for independent factors associated with local recurrence.

Only four patients had significant missing data insofar as histopathological data were missing. They were subsequently excluded from the final analysis. There are no recurrences in these four patients at the time of publication.

Statistical analysis was performed using STATA statistical software (version 16.0, STATA Corp) for data compilation, validation and analysis. Data analysis was performed between 01/08/2021 and 01/10/2021.

## Results

Out of 1000 patients, 996 evaluable patients who received full dose APBI through IOERT between 23 February 2010 and 08/10/2019 were identified. Patient demographics, tumour characteristics and treatment are summarized in Table 1. The median age was 61.5 (range 40–89) years. Median follow-up was 71.9 months according to the reverse Kaplan–Meier method. As of 1 August 2020, this study has a 97.0% complete follow-up using the completeness measure proposed by Clark et al. [27]

**Table 1:** Patient and tumour characteristics

Number of patients	996
Age, years	
< 50	127 (13%)
50–59	297 (30%)
60–69	343 (34%)
> 70	229 (23%)
Median applicator size	55 mm
Median applicator size/tumour size	4.6
Pathological tumour size	
≤ 1.0 cm	415 (42%)
> 1.0–1.5 cm	371 (37%)
> 1.5–2.0 cm	181 (18%)
> 2.0 cm	29 (3%)
Histology	
Ductal	952 (96%)
Other	20 (2%)
Lobular	15 (1%)
Mixed	9 (1%)
Multifocal breast cancer	33 (3%)
Grade	
1	415 (42%)
2	380 (38%)
3	201 (20%)
Oestrogen receptor status	
Positive	898 (90%)
Negative	98 (10%)
Progesterone receptor status	
Positive	812 (82%)
Negative	182 (18%)
Unknown	2
Proliferative index (Ki-67)	
< 14%	664 (67%)
14–20%	162 (16%)
> 20%	170 (17%)
HER2 status	
Negative	927 (93%)
Positive	69 (7%)
Molecular subtype	
Luminal A-like	583 (59%)
Luminal B-like	316 (32%)
HER2 positive	22 (2%)
Triple negative	75 (8%)
Perineural invasion	14 (1%)
Lymphovascular invasion	66 (7%)
Adjuvant treatment^#^	
None	29 (3%)
Hormone therapy alone	733 (74%)
Chemotherapy alone	80 (8%)
Hormone therapy and chemotherapy	154 (15%)
pN	
N0	960 (96%)
N1mic	21 (2%)
N1a	14 (1%)
N2	1

^#^This represents intention to treat, not treatment completion

Some percentages do not total 100% because of rounding

During follow-up, 37 ipsilateral breast tumour recurrences (IBTR) occurred with an incidence rate estimated at 2.7% (95% CI 1.8–4.0) at five years and 3.5% (95% CI 2.4–5.2) at median follow-up. Of those 37 recurrences, 16 were in the same breast quadrant. Additionally, there were 22 distant metastases (of which six had prior local recurrence) and five lymph node metastases (of which one had prior local recurrence). Overall relapse-free survival was estimated to be 92.3% (95% CI 90.8–94.4) at five years and 90.3% (95% CI 87.9–92.2) at median follow-up. The rate of distant metastasis occurrence was 1.7% (95% CI 1.0–2.9) and 2.2% (95% CI 1.3–3.5), respectively, at the five-year mark and at median follow-up. Likewise, overall survival was as high as 95.9% (95% CI 94.3–97.1) at five years and 94.4% (95% CI 92.4–95.9) at median follow-up.

There were 60 (6.0%) deaths among our cohort. Of those, 12 (1.2%) were attributable to breast cancer.

Table 2 summarizes the results of our multivariate competing risk analysis of factors associated with IBTR. Patients meeting institutional criteria for BRCA genetic assessment (these criteria are close to the 2010 NCCN guidelines [28]) and with a multifocal breast cancer were associated with a significantly increased risk of IBTR. Tumour size is on the verge of statistical significance for increased risk of IBTR. Age, lymphovascular involvement, perineural invasion, tumour grade and molecular subtypes were not associated with IBTR.

**Table 2:** Competing risk analysis of factors associated with IBTR

	SHR	95% CI	*P* value
Genetic testing criteria met^a^	2.92	1.18–7.23	**0.020**
Age (years)			
< 50	1.00	Ref	
50–59	0.47	0.17–1.32	0.153
60–69	0.47	0.16–1.31	0.149
≥ 70	0.83	0.32–2.18	0.708
T stage group			
pT1a and pT1b	1.00	Ref	
pT1c and pT2	2.24	0.99–5.06	0.053
Molecular subtype			
Luminal A-like	1.00	Ref	
Luminal B-like	0.95	0.43–2.13	0.907
HER+	2.96	0.79–11.0	0.106
Triple negative	1.98	0.70–5.59	0.197
Multifocal breast cancer	3.97	1.36–11.5	**0.011**
Lymphovascular involvement	1.07	0.30–3.75	0.921
Perineural invasion	2.20	0.37–13.0	0.384

Bold was used to illustrate results with p-values below the significance cutoff of 0.05

^a^A patient is said to meet genetic testing criteria after a trained clinical geneticist confirms there is an indication for genetic testing

Acute treatment toxicity remained low, with 15 (1.5%) hematomas (none requiring subsequent surgery), eight (0.8%) infections, nine (0.9%) wound dehiscence, six (0.6%) patients having both an infection and wound dehiscence, ten (1%) patients with delayed uncomplicated wound closure, and one (0.1%) case of wound necrosis. Late toxicities and cosmetic results are reported in Table 3. Late toxicities are not given for the entire set of patients given that those assessments were progressively discontinued.

**Table 3:** Late toxicity and cosmetic results

Oedema	
No oedema	853 (99.7%)
Minimal oedema	3 (0.3%)
Data not reported	140
Skin pigmentation	
No skin discoloration	855 (100%)
Not reported	141
Dimpling	
No dimpling	854 (99.8%)
Mild dimpling	2 (0.2%)
Not reported	140
Scar prominence	
Scar unapparent	822 (96.3%)
Scar apparent	17 (2.0%)
Moderately apparent scar	15 (1.8%)
Not reported	142
Breast asymmetry	
No effect	496 (58.1%)
Minimal asymmetry	254 (29.8%)
< 1/3	84 (9.9%)
> 1/3	19 (2.2%)
Not reported	143
Overall cosmetic result	
Excellent	492 (57.5%)
Good	260 (30.4%)
Fair	91 (10.6%)
Poor	12 (1.4%)
Not reported	141

Some percentages do not total 100% because of rounding

## Discussion

First, a significant part of our patient population is aged 65 or older. These patients would also fit all criteria for completely omitting breast radiotherapy as attempted in clinical trials [29–31]. These trials showed that omitting WBI resulted in no difference in overall survival in this select population. Yet, a significant increase in IBTR was demonstrated in all trials with a risk difference of about 6% at 10 years in favour of WBI. Thereafter, some guidelines were modified to include RT omission in elderly patients [4, 32]. Some might thus think that RT for elderly patients with early stage breast cancer is no longer needed. However, it should be noted that while WBI is most likely an overtreatment for most of this patient population, APBI was never tested as a comparator in these clinical trials. We would argue that APBI and IORT techniques in particular could well become the best treatment modality for these patients. Indeed, these immediate, one-shot RT treatments still allow for a strong reduction in treatment burden while yielding strong IBTR risk reduction as discussed below.

Our results are aligned with those of the main external and brachytherapy APBI trials [11, 13–15, 33–35]. While IMPORT LOW has lower five-year IBTR rates than our study, the populations are not similar thus precluding any direct comparison. For example, 23% of our trial population received chemotherapy, 20% had grade 3 tumours against only 5% and 10%, respectively, in IMPORT LOW. The same goes for the GEC-ESTRO trial population. Additionally, we have a relatively similar IBTR rate to both RAPID and the Florence trial, which had populations that more closely resembled our study population. This is particularly significant given that contrary to external APBI, IOERT has insignificant irradiation of organs at risk.

Compared to the populations of the TARGIT-A and ELIOT trials, our study population has far fewer node positive tumours of a size greater than 2 cm or lobular breast cancers [22, 36, 37]. This was expected given the inclusion criteria of this study. Population grade was comparable to both trials. There were more Luminal B-like tumours in ELIOT than in our study. Although there is no molecular subtype data available for TARGIT, surrogate markers (such as ER/PR positivity and tumour grade) are somewhat similar. We included younger patients than both studies given enrolment was open to patients as young as 40.

Interestingly, the results of this study closely resemble those of the TARGIT-A clinical trial IORT arm, one of the two main trials to have compared IORT to WBI. TARGIT-A outlined an IBTR 5-year recurrence risk of 3.3% and overall survival of 96.1%. These results are comparable to the 2.7% and 95.9% rates found by this study [38]. Although the intraoperative radiotherapeutic technique used in our study is different to the one used in TARGIT-A and any comparison should therefore be made carefully, it is interesting to note that similar IBTR and OS outcomes speak in favour of our technique being as safe and efficacious as that of the TARGIT trial when applied to selected populations. It should also be noted that a 2.7% IBTR rate at five years is well within the acceptable non-inferiority margin used in clinical trials comparing APBI with WBI.

Our study also seems to have yielded better IBTR rates than those found in the ELIOT trial (4.4% vs 2.7%). As the IORT technique used in this study is the same as the one in used in the ELIOT trial (IOERT), this is of particular interest. Crucially, differences in lymph node metastasis (1% vs 0.2% in our study) and in distant metastasis (respectively 4.5% vs 1.7%), 5-year rates can also be noted.

We have multiple hypotheses to explain these differences in outcome:

First, the ELIOT trial began enrolment more than a decade before the first patients in our study were treated. During that period, chemotherapy treatment guidelines changed, adopting, among others, taxanes and Trastuzumab into regular chemotherapy regimens for breast cancer. The additional efficacy of these new chemotherapy regimens might have contributed to better results in more aggressive breast cancer subtypes/grade and brought IBTR risk closer to baseline favourable subtype/low grade breast cancer in our study.

Second, investigators in the ELIOT trial enrolled patients with more advanced tumours than in our cohort. Most notably, ELIOT enrolled larger size (up to 2.5 cm), node positive cancers in their trial. Out-of-quadrant dissemination and subsequent IBTR could therefore be more likely. Similarly, lobular breast cancers were enrolled although they are more likely to be multifocal and therefore again risk increasing the IBTR rate [39].

Third, although our radiotherapy technique is similar to ELIOT’s, it is highly operator-dependent. Many variables account for a successful IOERT treatment. Most notably, the treatment critically relies on applicator size and surgical technique to bring the bordering breast tissue inside the irradiation field [40, 41]. The median applicator size in this study is 55 mm compared to 40 mm in ELIOT [42]. In fact, given that the median tumour size in this study is smaller than in the ELIOT trial, the difference should be even greater. Table 4 summarizes the different applicator sizes of both ELIOT and our study. Systematic applicator under sizing has a profound impact on radiotherapy treatment. Out of foci recurrence, for example, has been associated with smaller applicator sizes [43, 44]. In the case of this study, a 15 mm increase in applicator size increases the treated surface by a factor of about 1.89. We would argue this is probably the most important difference between the two studies, and it probably explains most of the difference between ELIOT’s higher IBTR rate and this study.

**Table 4:** Distribution of applicator sizing in ELIOT and this study

Applicator size	ELIOT (%)	Jules Bordet cohort (%)
30	0.3	0
35	0	0.1
40	51.5	0
45	0	6.1
50	37.4	26.3
55	0	38.7
60	10	26.8
65	0	2
70	0.2	0
80	0.2	0

Much thought had gone into creating a protocol for choosing an appropriate applicator size for patients. We based ours on studies by Holland and Faverly et al. [45, 46] These studies focused on post-mastectomy specimens in the 1980s and showed that a significant number of patients had residual tumour at more than 1 cm from tumour edge. The number of patients having residual tumour tissue dropped by almost half if the surgical margin was increased to 3 cm. We therefore chose—albeit somewhat arbitrarily—to have a total tumour margin of at least 3 cm. This was usually composed of a 1 cm surgical microscopic tumour-free margin and a radiotherapy margin of at least 2 cm. We also introduced a proportional increase in the radiotherapy margin size according to tumour size (Additional file 1: Appendix A). For a 5-mm tumour, for example, the radiotherapy margin was at 2.25 cm and the total margin (surgical + radiotherapy) at 3.25 cm. In the case of a 20-mm tumour, however, these margins would increase to 3 and 4 cm, respectively (Additional file 1: Appendix A).

ELIOT trialists had undertaken a stratified post hoc analysis of factors associated with IBTR [21]. The trial showed on univariate analysis that among others, Luminal B and triple negative molecular subtypes and tumour grade were very strong factors associated with IBTR. Our study, however, has found that while IBTR is strongly associated with patients meeting genetic testing criteria and presenting with plurifocal breast cancer (factors we therefore consider to be relative contraindication to IOERT), grade and molecular subtype were not significantly related to IBTR. In all likelihood, there probably is a slightly increased risk of IBTR with more aggressive cancer subtypes, with increased tumour grade, or with proliferative index, but all these factors are probably also responsible for increased tumour size, and this multicollinearity would perhaps explain part of the non-significance of those variables. As bigger tumours are more likely to have out-of-quadrant foci, tumour size being on the verge of significance in our multivariate analysis therefore seems quite self-explanatory.

Given the results of this study, we hypothesize that tumour size is still a very significant factor of IBTR and that rather than contraindicating some aggressive molecular subtypes or tumour grades, it would be more beneficial to take into account both tumour size and then secondary tumour characteristics in potential guidelines [47, 48]. It should be noted that the post-hoc analyses used in the ELIOT trial to try to find factors associated with IBTR were just exploratory in nature, as were those underpinning our study. Moreover, factors found to be linked to IBTR are very inconsistent across different APBI trials with some finding no link between molecular types or grades and IBTR [16, 49]. Thus, guidelines based on these analyses should always be prospectively assessed.

One main study limitation is that cosmetic evaluations were progressively discontinued given the difficulty in collecting quality data. In total, about 855 patients benefited from a summary long-term skin toxicity and cosmesis assessment. Overall treatment for evaluated patients was demonstrated to be well-tolerated for acute and late toxicity as well as breast cosmesis in keeping with the previously published literature [17, 50–52].

This study provides real-life data proving that IOERT using our inclusion criteria yields a low IBTR rate similar to the successful TARGIT IORT clinical trial. It is therefore the conclusion of this study that IOERT guidelines are probably too restrictive and that they could be opened up to allow inclusion of all molecular subtypes/tumour grade, with some restrictions based on tumour size.

Most importantly, this study seeks to highlight the fact that IOERT results in extremely low irradiation to organs at risk due to pectoral wall shielding. Given the significant improvements in breast cancer survival in the past decades, the authors share Vaidya et al.’s opinion that reducing treatment toxicity is now of the upmost importance [18]. Since radiotherapy-related morbidity and mortality is mediated by the irradiation of organs at risk, it is understood that drastically reducing the dose given to those organs with IOERT will reduce non-subcutaneous tissue-related toxicity [19, 20]. It follows that when comparing IOERT to WBI, additional local recurrences can be tolerated, if the patient wills it, as long as they do not affect breast cancer associated mortality. This is especially true as local recurrence does not seem to be as strong an indicator of poor prognosis in IORT as it has been in WBI [53]. Such a trade-off is therefore justified in light of the net gain in overall mortality that can be expected on the basis of lower non-breast cancer mortality [18]. We are of the opinion that overall we should take care not to harm patients by overtreating them with WBI when other IORT treatments are available and have demonstrably no negative impact on breast-cancer mortality.

Nevertheless, we must stress the importance of appropriate and exhaustive patient workup with MRI imaging by a specialized breast radiologist, timely pathological examination of the core biopsy and tumour frozen section as key elements contributing to these good results. If, at any point during patient workup, there was some lingering doubt about IOERT suitability, we would not proceed with the technique.

## Supplementary Information

**Additional file 1.** Supplementary information on the surgical and radiotherapy techniques.

## Data Availability

The datasets analysed during the current study are not publicly available as of yet but are available from the corresponding author on reasonable request.
