# Supplementary material for: Intraoperative electron radiotherapy in early invasive ductal breast cancer: 6-year median follow-up results of a prospective monocentric registry
Source: Breast Cancer Res. 2022 Nov 23;24:83. doi: 10.1186/s13058-022-01582-4 (PMC9685863; doi:10.1186/s13058-022-01582-4)
Supplement: Supplementary file 1 — Additional file 1. Supplementary information on the surgical and radiotherapy techniques. [file 13058_2022_1582_MOESM1_ESM.docx]

**Appendix A: Supplementary information on the surgical and radiotherapy techniques**

**This information is mainly based on a previous article of the author**^1^**.**

The surgical procedure began with removal of the sentinel lymph node(s) (SLN), which were identified with a gamma probe. A lumpectomy was then performed, via an elliptical skin incision made directly over the tumour. This allows introducing the radiation applicator through the incision and offers optimal control of the anterior surgical margin. It also makes it possible to mobilize the target glandular tissues surrounding the lumpectomy bed for radiation. The tumour was removed in one piece with a 1 to 2 cm safety margin extending posteriorly, where possible, to the aponeurosis of the pectoralis major muscle. The surgical specimens including the sentinel nodes were sent to the pathology laboratory for intraoperative analysis of tumour size, surgical resection margins, and SLN malignancy. If the histological criteria were met (pT1, safe margins ≥1 mm (except posteriorly in the case of a tumour bordering the pectoral aponeurosis in which case the aponeurosis should be removed to ensure safe margins)), pN0), the target tissue was dissected from the underlying pectoralis major aponeurosis in order to be able to put a protective shield in place.


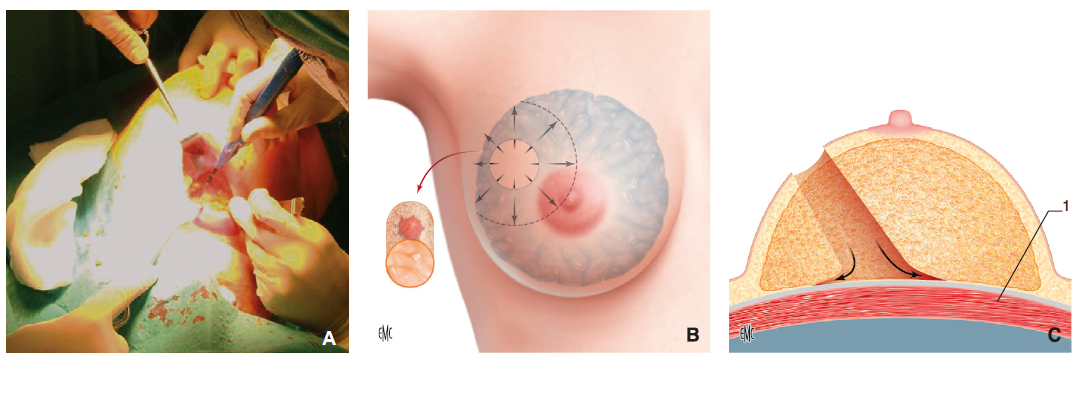


Figure 1 Pectoral Aponeurosis dissection (courtesy Elsevier Masson)

A : Operating room view of the aponeurosis dissection

B and C : Tumourectomy piece and subsequent dissection of the pectoral aponeurosis. The arrows represent the extent and direction of the deep tissue dissection.

The shield consisted of 4 mm of steel and 4 mm of aluminium (the steel is closer to the pectoral aponeurosis and the aluminium faces the opposite way) so that all the electrons were intercepted by the steel and the electrons back-scattered by the steel were blocked by the aluminium. The total thickness of the shield is equivalent to more than 42 mm of water. For 9 MeV electrons, as this energy requires a bolus of at least 5 mm of thickness to ensure that the target surface receives the prescribed dose, the combination of bolus and shield completely stops the electrons, independently of the treated target thickness. For 12 MeV electrons, which are only used if the thickness to be treated is bigger than 29 mm, the combined equivalent thickness of treated tissues and shield is thick enough to completely stop the electrons.

The gland was then dissected of the subcutaneous fat and skin and as much of the breast tissue potentially containing residual microscopic cancerous foci as possible brought into the tumour bed: these tissues were sutured over the shield.

The shield generally used was 15 mm larger in diameter than the applicator whose diameter was already 40 mm larger than the tumour itself (size of the breast permitting), in order to create a 20 mm safety margin around the tumour bed.

This surgery is done under the cover of perioperative antibioprophylaxis by a 2^nd^ generation cephalosporin.


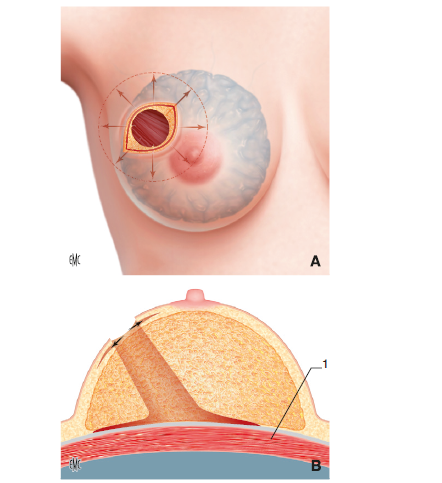


Figure 2 Superficial Subcutaneous tissue dissection (courtesy Elsevier Masson)

A : Frontal view

B : Sectional view, the arrows represent the extent and direction of the subcutaneous dissection

1 : Pectoral muscle


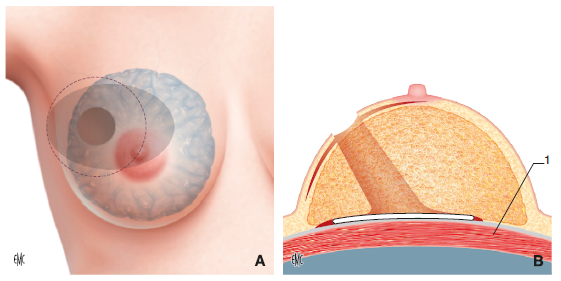


Figure 3 Pectoral shielding placement (courtesy Elsevier Masson)

A : Frontal view

B : Sectional view with the shield in position on top of the pectoral muscle

1 : Pectoral muscle


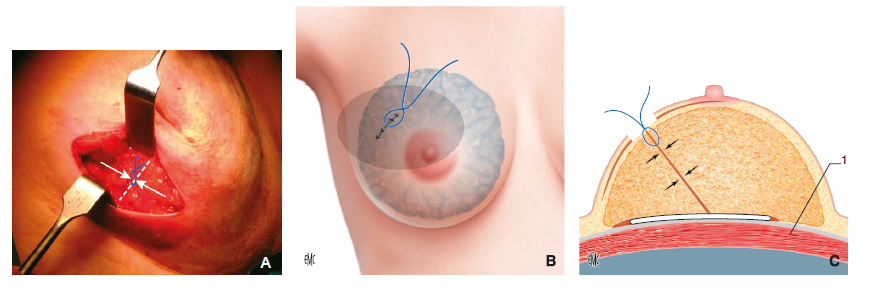


Figure 4 Breast gland suture prior to irradiation (courtesy Elsevier Masson)

A : Operating room view

B and C : Frontal and sectional view. The arrows represent breast tissue mobilisation towards the tumour bed


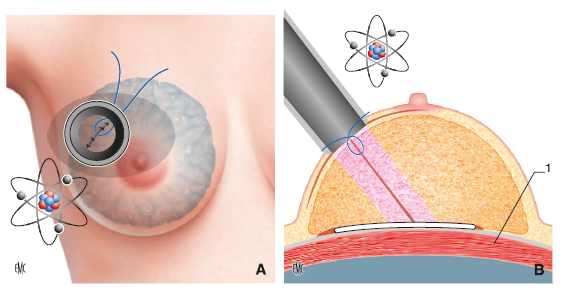


Figure 5 : Tissue irradiation (courtesy Elsevier Masson)

A : Frontal view

B : Sectional view.

*2.3. Radiation Therapy.*

All patients were treated with electrons generated by an IntraOp (Mobetron) dedicated mobile accelerator. The diameter of the cylindrical applicators available varied from three to 10 cm, in 0.5 cm increments. Our IOERT PTV policy was adapted to the tumour size. As a rule, the field diameter used was at least 40 mm bigger than the pathological tumour diameter and whenever feasible, theoretical diameters were rounded up to the bigger applicator. For example, for a 6 mm tumour, we would add 4 centimetres to obtain a 46 mm treatment diameter. This would then be rounded up to a 50 mm applicator and so on so forth.

The dose delivered was 21 Gy, prescribed over the 90% isodose line.

The 90% isodose line diameter at dmax is slightly smaller than the nominal field diameter (from 6 to 9 mm smaller, depending on field size and energy). Taking that into account, we have around the tumour bed a treated volume whose diameter is at least 36 mm for pT1a tumours, 41 mm for pT1b, and 51 mm for pT1c tumours. The applicator extremity was either flat or bevelled (15° to 30°). Electron energies of 6, 9, and 12 MeV were available. The 9 MeV was the energy the most frequently selected (range 6–12 MeV). A 5 or 10 mm bolus was used either to increase the entrance dose to at least 90% or to decrease the total electron range in the patient. Energy was determined in function of the maximum thickness of the target tissue, in order to have the 90% isodose depth greater than the maximum target thickness. The maximum target thickness was simply measured by introducing a needle into the tissue down to the protective shield placed over the muscle. Beam calibration was performed on the treatment day for quality control purposes. For the first 200 patients, the position of the protective shield was controlled intraoperatively by inserting a needle into the gland at a tangent to the applicator and ensuring that it “hit” the shield. Afterwards a new technique was developed, 3 long steel rods were screwed into the shield so that their free end stuck out of the patient. The applicator was then positioned at the centre of these 3 rods thus ensuring complete pectoral wall shielding. During radiation, a digital radiograph was positioned underneath the patient on the Mobetron beam-stopper to maintain an exact record of the shield’s position relative to the applicator.

1. Philippson C, Simon S, Vandekerkhove C, et al. Early Invasive Cancer and Partial Intraoperative Electron Radiation Therapy of the Breast: Experience of the Jules Bordet Institute. *Int J Breast Cancer*. 2014;2014:1-6. doi:10.1155/2014/627352
